# *SF3B1* mutation generates novel peptides from long non-coding RNAs

**DOI:** 10.64898/2026.10.15.759235

**Published:** 2026-10-16

**Authors:** Supriyo Bhattacharya, Mike Fernandez, Yiming Wu, Meiling Jin, Patrick Pirrotte, Krystine Mansfield, Tae Hyuk Kang, Yuqi Zhao, Nicole Lammana, Xiwei Wu, Lili Wang

## Abstract

While mRNA alternative splicing caused by somatic mutation in *SF3B1* is well-characterized, long non-coding RNAs (lncRNAs) impacted by this mutation are completely unknown. Here, we report long-read sequencing of full-length transcripts from cell lines with and without SF3B1 mutation. We present ALLR (Annotation of human lncRNAs using Long-Read sequencing), a computational workflow to identify and quantify lncRNAs, and to annotate their potential roles in impacting cellular functions. Harnessing matched short-read RNA sequencing and proteomic data, we demonstrate that *SF3B1* mutation generates thousands of novel lncRNAs with diverse levels of alternative splicing. Moreover, *SF3B1* mutation-associated lncRNAs produce translatable peptides with putative functions, immunogenicity, and potential to bind proteins with impact on cellular signalling networks. 47 lncRNAs could also be detected in primary chronic lymphocytic leukemia (CLL) samples and are associated with significant risks towards adverse disease prognosis, in conjunction with other known CLL risk factors. Our work demonstrates the utility of long-read sequencing for lncRNA research and provides a new territory of functional RNA molecules that SF3B1 mutation may use to impact cancer.

**GRAPHICAL ABSTRACT:**

## Full Text Availability

The license terms selected by the author(s) for this preprint version do not permit archiving in PMC. The full text is available from the preprint server.

